# How is cognition in subthalamic nucleus deep brain stimulation Parkinson’s disease patients?

**DOI:** 10.1590/1980-57642018dn13-040002

**Published:** 2019

**Authors:** Eduarda Naidel Barboza e Barbosa, Helenice Charchat Fichman

**Affiliations:** 1 Master, Pontifical Catholic University of Rio de Janeiro (PUC-Rio), Rio de Janeiro, RJ, Brazil.; 2 Professor, Pontifical Catholic University of Rio de Janeiro (PUC-Rio), Rio de Janeiro, RJ, Brazil.

**Keywords:** Parkinson’s disease, deep brain stimulation, subthalamic nucleus, cognition, doença de Parkinson, estimulação cerebral profunda, núcleo subtalâmico, cognição

## Abstract

The impairments in cognitive functions such as memory, executive function, visuospatial skills and language in Parkinson’s disease (PD) are drawing increasing attention in the current literature. Studies dedicated to investigating the relationship between subthalamic nucleus deep brain stimulation (STN-DBS) and cognitive functioning are contradictory. This systematic review aims to analyze the impact on the cognitive functioning of patients with PD and STN-DBS. Articles published in the 2007-2017 period were retrieved from the Medline/Pubmed databases using PRISMA criteria. The analysis of 27 articles revealed many conflicting results, precluding a consensus on a cognitive functioning standard and hampering the establishment of a neuropsychological profile for PD patients who underwent STN-DBS surgery. Further studies investigating this relationship are needed.

## Resumo

As deficiências nas funções cognitivas, como memória, função executiva, habilidades visuoespaciais e linguagem na doença de Parkinson (DP), estão cada vez mais chamando a atenção na literatura atual. Estudos dedicados a investigar a relação entre a estimulação cerebral profunda do núcleo subtalâmico (ECP-NST) e o funcionamento cognitivo são contraditórios. Esta revisão sistemática tem como objetivo analisar o impacto no funcionamento cognitivo de pacientes com DP e ECP-NST. Os artigos foram coletados nas bases de dados Medline / Pubmed publicadas no período de 2007-2017, utilizando os critérios do PRISMA. Após a análise de 27 artigos observou-se muitos resultados opostos, não sendo possível convencionar um padrão de funcionamento cognitivo o que dificulta o estabelecimento de um perfil neuropsicológico para pacientes com essa doença que foram submetidos à cirurgia de ECP-NST, sendo necessários mais estudos.

The diagnosis of PD is performed using clinical criteria by trained professionals, such as neurologists. These criteria are based on the identification of clinical manifestations and pure motor symptoms. Patients with PD present, in addition to motor impairments, non-motor impairments manifesting as a variety of neuropsychiatric symptoms,^1^^,^ 2 changes in sleep, behavior and cognition,^3^^,^ 4 and which may lead to dementia.^5^^,^ 6

The impairments in cognitive functions, such as memory, executive function, visuospatial skills and language in PD, are drawing increasing attention in the current literature.^6^ One in three patients with PD presents cognitive impairment at the time of (or soon after) diagnosis, which progressively worsens and may even cause dementia in the later stages of the disease.^7^

Since 1940, surgical treatment of PD has been performed. More recently, since 1998, ablation has given rise to deep brain stimulation (DBS) targeting the subthalamic nucleus (STN) or globus pallidus internus (GPi).^5^^,^ 8 The target most chosen by centers performing the surgery is the STN due to the possibility of decreasing drug doses and, consequently, reducing adverse effects.

The literature points to evident motor and QoL improvement after DBS in patients with PD. However, studies dedicated to investigating the relationship between STN-DBS and cognitive functioning are controversial, and further studies investigating this relationship are needed.

In this context, the investigation of the cognitive effects of STN-DBS in PD becomes paramount. The objective of this study is to analyze the effects of subthalamic nucleus (STN) DBS on the cognition of PD patients through a systematic review. The Preferred Reporting Items for Systematic Review and Meta-Analyzes (PRISMA) Checklist was employed.

## METHODS

The systematic review is a type of scientific research that aims to gather, critically evaluate and conduct a synthesis of multiple primary studies.^10^

### Bibliographic survey

We designed a systematic review of the literature according to the Preferred Reporting Items for Systematic Review and Meta-Analyzes (PRISMA) criteria. The following terms were used: “Deep Brain Stimulation”, “DBS”, “Cognitive Functions” and “Parkinson Disease” with the Boolean operator “AND”. We selected scientific papers published in English between January 2007 and January 2017, with comparative clinical trials in humans, on the Medline/Pubmed databases. Articles published before 2007, systematic reviews, case studies, books chapters and studies using animals were excluded.

### Studies selection

**Figure 1:**
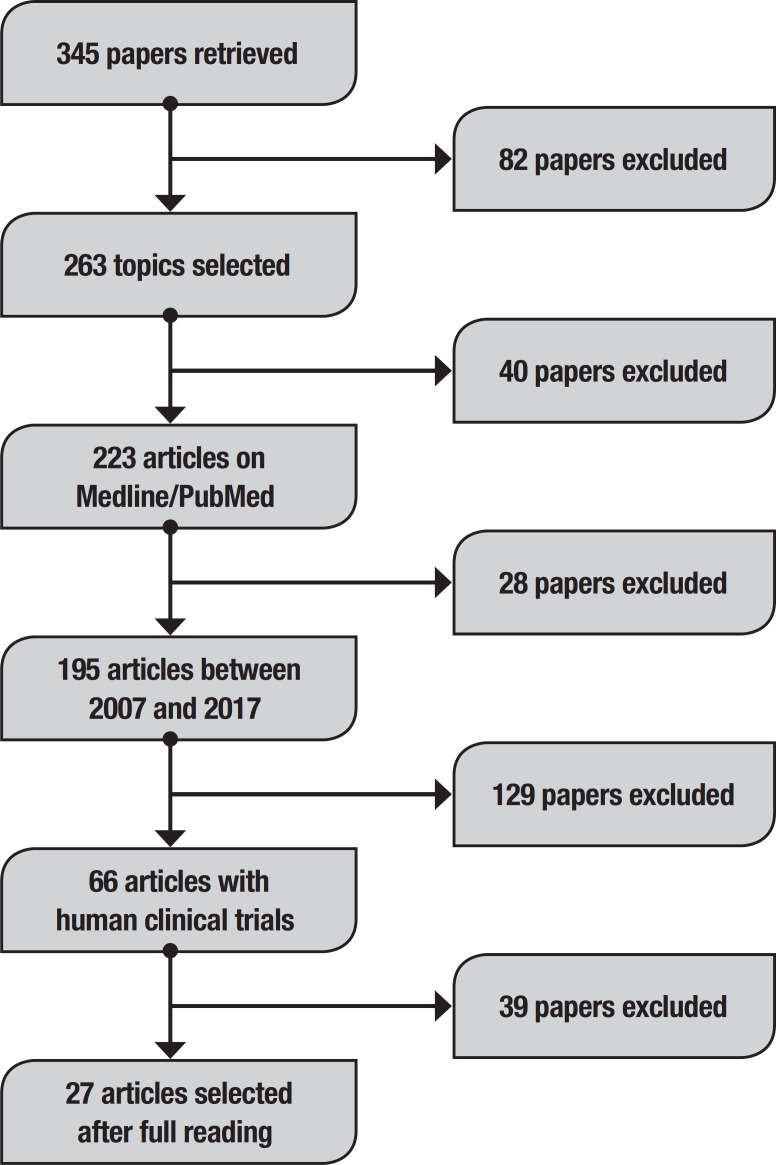
Article search flow diagram.

Initially, this method retrieved 345 papers (Figure 1). To refine the research, the following topics were selected: “Parkinson’s Disease”, “Subthalamic Nucleus”, “Deep Brain Stimulation”, “DBS”, “Cognition” (263), published on the Medline/Pubmed databases (223) between 2007 and 2017 (195). For the papers selected, a title and abstract analysis was performed manually to consider only studies with human clinical trials (66). Literature reviews and case studies were excluded, as were articles containing problems in the methodology, such as absence of: (a) inclusion and exclusion criteria; (b) complete assessment protocol; and (c) pre or post-surgery assessment (27). The researchers selected the articles independently: they considered suitable studies that: (a) evaluated cognition of PD patients with STN-DBS; (b) presented the instruments and domains evaluated; and (c) reported pre and post-surgical results of articles. 

## RESULTS

The final list of included articles that met the study criteria, in ascending order of year, together with objectives and results, is given in Table 1. A list of studies, grouped according to the effects of DBS on specific cognitive domains, with neuropsychological tasks (carried out in each study assessed) is given in Table 2.

**Table 1:** List of articles included in the systematic review.

Name	Year	Objective	Result
[1] Cilia et al. Brain networks underlining verbal fluency decline during STN-DBS in Parkinson's disease: An ECD-SPECT study.	2007	To assess changes on evaluation after DBS-STN and their possible correlation with the cognitive result related to the frontal lobe.	Patients with STN-DBS improved motor symptoms and reduced medications, but selectively declined in category fluency.
[2] Klempírová et al. Deep brain stimulation of the subthalamic nucleus and cognitive functions in Parkinson's disease.	2007	To evaluate how STN-DBS affects cognitive functions.	Patients treated by STN-DBS tend to worsen in executive functions and logical memory.
[3] Castelli et al. Apathy and verbal fluency in STN-stimulated PD patients.	2007	To evaluate apathy and its relationship with verbal fluency tasks in patients with PD who underwent STN-DBS.	The results suggest that STN-DBS does not necessarily induce apathy, even if individual patients show moderate postoperative worsening of apathetic symptoms.
[4] Heo et al. The effects of bilateral Subthalamic Nucleus Deep Brain Stimulation (STN-DBS) on cognition in Parkinson disease.	2008	To research STN-DBS effects on cognition and mood.	Bilateral STN-DBS did not lead to a significant overall deterioration in cognitive function. However, it has small, long-term detrimental impacts on memory and frontal lobe function.
[5] Witt et al. Neuropsychological and psychiatric changes after deep brain stimulation for Parkinson's disease: a randomised, multicentre study.	2008	To evaluate DBS neuropsychiatric consequences in patients with PD.	STN-DBS does not reduce overall cognition or affectivity, although there is a selective decrease in frontal cognitive functions and an improvement in anxiety in patients after treatment, changes not affecting improvements in quality of life.
[6] Alberts et al. Bilateral subthalamic stimulation impairs cognitive-motor performance in Parkinson's disease patients.	2008	To determine the effects of unilateral and bilateral STN-DBS on upper extremity motor function and cognitive performance under single and double-task conditions in patients with advanced PD.	Significant declines in cognitive and motor function under modest dual-task conditions with bilateral , but not unilateral STN-DBS.
[7] Lueken et al. Impaired performance on the Wisconsin Card Sorting Test under left- when compared to right-sided deep brain stimulation of the subthalamic nucleus in patients with Parkinson's disease.	2008	To evaluate whether changes in performance on executive tasks after chronic DBS may be predominantly associated with stimulation of only one hemisphere.	The STN is not only involved in motor control, but also participates in functions of the cognitive domain. All patients had a significant improvement in motor symptoms postoperatively. Selected aspects of executive task performance were compromised under left - when compared to right-sided stimulation.
[8] Zangaglia et al. Deep brain stimulation and cognitive functions in Parkinson's disease: A three-year controlled study	2009	To evaluate DBS cognitive and behavioral effects.	Verbal fluency worsening after DBS, but relatively safe surgery from a cognitive point of view, since short-term worsening of front-executive functions was transient.
[9] Williams et al. Deep brain stimulation plus best medical therapy versus best medical therapy alone for advanced Parkinson's disease	2010	To evaluate whether surgery and best medical therapy improved self-reported quality of life more than best medical therapy alone	After 1 year, surgery and best medical therapy improved patient self-reported quality of life more than best medical therapy alone in patients with advanced PD, constituting clinically meaningful differences.
[10] York et al. Relationship between neuropsychological outcome and DBS surgical trajectory and electrode location	2009	To observe whether differences in position of electrode and surgical trajectory of DBS can lead to differential neuropsychological outcome.	Cognitive and emotional changes after 6 months of bilateral STN-DBS may be related to surgical trajectory and positioning of electrodes.
[11] Daniels et al. Risk factors for executive dysfunction after subthalamic nucleus stimulation in Parkinson's disease	2010	To evaluate baseline parameters that contribute to deterioration of cognitive functioning after DBS.	Surgical procedure, exact placement of electrode or postoperative management might be more relevant for a decline in executive functioning after STN-DBS, in addition to factors such as age, high levodopa dosages and high scores on the UPDRS III axial subscore in OFF state.
[12] Castelli et al. Neuropsychological changes 1-year after subthalamic DBS in PD patients: A prospective controlled study	2010	To investigate the neuropsychological effect of STN-DBS in patients with advanced PD.	Phonemic verbal fluency declined one year after STN-DBS, while the other cognitive domains did not change significantly. Only 4 subjects had significant cognitive decline 1 year after surgery.
[13] Fasano et al. Motor and cognitive outcome in patients with Parkinson's disease 8 years after subthalamic implants	2010	To assess long-term PD patients undergoing STN-DBS for 8 years: long-term motor outcome of symptoms that improve in the short and medium-term with STN-DBS; identification of predictors of long-term motor outcome; and long-term cognitive and behavioral outcome.	STN-DBS is a safe procedure regarding cognitive and behavioral morbidity over long-term follow-up. However, the global benefit decreases later in the course of the disease due to the progression of PD and to the appearance of stimulant-resistant medications and symptoms.
[14] Van Wouwe et al. Deep Brain Stimulation of the Subthalamic Nucleus Improves Reward-Based Decision-Learning in Parkinson's Disease	2011	To investigate the effect of STN-DBS on reward-based learning in patients diagnosed with PD.	DBS cognitive effects benefited a subset of relatively younger patients with relatively shorter disease duration in daily-life association-learning situations.
[15] Israeli-Korn et al. Subthalamic Nucleus Deep Brain Stimulation Does Not Improve Visuo-Motor Impairment in Parkinsons Disease	2013	To evaluate how STN-DBS affects visuo-motor coordination in patients with PD.	Clinically-measured "low-level" motor function responds to STN-DBS, but cognitive and "high-level" motor functions related to VMC may not respond to STN-DBS.
[16] Kim et al. Initial cognitive dip after subthalamic deep brain stimulation in Parkinson disease	2013	To examine whether the rate of change in global cognitive functioning during the initial 6 months after STN-DBS differed from the mean 6-month change that occurred between 6 and 36 months after surgery.	The decline in global cognitive function was faster in the first 6 months after surgery, compared to a 6-month period between 6 and 36 months post-surgery.
[17] Yágüez et al. Cognitive predictors of cognitive change following bilateral subthalamic nucleus deep brain stimulation in Parkinson's disease	2014	To specifically establish a detailed neuropsychological profile before and after STN-DBS and identify any pre-surgical cognitive profile that can predict cognitive outcomes after stimulation.	Non-dementia patients with mild impairment in both general intellectual functions and list learning, may be at a greater risk of decline in other aspects of verbal memory after STN-DBS.
[18] Asahi et al. Impact of bilateral subthalamic stimulation on motor/cognitive functions in Parkinson's disease	2014	To systemically assess the impact of bilateral STN-DBS on motor and cognitive functions in patients with PD.	Bilateral STN-DBS can significantly improve cognitive function in a given subgroup of patients whose therapeutic effects on motor function are prominent.
[19] Rizzone et al. Long-term outcome of subthalamic nucleus DBS in Parkinson's disease: From the advanced phase towards the late stage of the disease?	2014	To report the results of a long-term follow-up of patients implanted with DBS bilaterally in two centers.	Despite the STN-DBS long-term safety and efficacy in PD, patients functionality worsened over time, mainly for the onset and progression of levodopa-resistant and non-motor symptoms.
[20] Houvenaghel et al. Reduced Verbal Fluency following Subthalamic Deep Brain Stimulation: A Frontal-Related Cognitive Deficit?	2015	To explore the mechanisms underlying DBS.	Cognitive slowdown and apathy seem to have a more decisive influence on the impairment of phonemic verbal fluency after DBS.
[21] Markser et al. Deep brain stimulation and cognitive decline in Parkinson's disease: The predictive value of electroencephalography	2015	To examine whether clinical recordings of EEG can be used to predict cognitive impairment in PD patients undergoing STN-DBS.	The GTE preoperative score can be used to identify patients with PD who are at high risk of developing cognitive impairment after STN-DBS surgery even though their preoperative cognitive status is normal.
[22] Pham et al. Self-Reported Executive Functioning in Everyday Life in Parkinson's disease after Three Months of Subthalamic Deep Brain Stimulation	2015	To compare self-reported daily executive functioning in patients with PD before and after three months of STN-DBS.	Patients with PD showed significant improvement in daily life executive functioning 3 months after surgery. Anxiety indexes decreased significantly while psychiatric symptoms, including apathy, remained unchanged. Only preoperative depressive mood had predictive value for the improvement of executive function and seems to prevent potentially favorable results from the STN-DBS in some aspects of the executive function.
[23] Tang et al. Evidence of improved immediate verbal memory and diminished category fluency following STN-DBS in Chinese-Cantonese patients with idiopathic Parkinson's disease	2015	To investigate neuropsychological effects of STN-DBS in Chinese-Cantonese patients with PD.	A diminished performance of verbal fluency was observed, on the other hand, an improvement in immediate verbal memory, besides anxiety level were demonstrated.
[24] Tremblay et al. The effects of subthalamic deep brain stimulation on metaphor comprehension and language abilities in Parkinson's disease	2015	To determine the effects of STN-DBS on the comprehension of metaphor and linguistic abilities such as lexical and semantic abilities.	STN-DBS had a significant beneficial effect on motor symptoms in PD, but this stimulation had no effect on metaphor comprehension or any other cognitive ability assessed in this study.
[25] Krishnan et al. The decade after subthalamic stimulation in advanced Parkinson's disease: A balancing act.	2016	To examine the long-term quality of life, motor and cognitive outcomes of bilateral subthalamic nucleus STN DBS and the pre-DBS factors that predict sustained motor benefits at or beyond 7 years from surgery.	Improvements in severity of motor fluctuations, stiffness, and tremor are the most enduring STN-DBS benefits, lasting a decade. However, these are offset by the higher levodopa requirement, and worsening cognitive and axial functions, bradykinesia and dyskinesias.
[26] Vonberg et al. Fabian. Deep Brain Stimulation of the Subthalamic Nucleus Improves Lexical Switching in Parkinson's disease Patients	2016	To outline the nature of verbal fluency dysfunction.	The STN-DBS group task performance was lower than that of healthy controls. In addition to affecting motor symptoms, surgery seems to influence the dynamics of cognitive procedures.
[27] Ventre-Dominey et al. Distinct effects of dopamine vs STN stimulation therapies in associative learning and retention in Parkinson disease	2016	To investigate and compare results of treatment with dopamine versus DBS in the ability of PD patients to acquire and maintain over the successive days their performance in visual working memory	While STN-DBS patients demonstrate more accurate and faster responses in the ON stage than in the OFF stage, regardless of the day of testing, patients using dopamine replacement therapy had more accurate and faster ON response compared to OFF during the first day of learning and then maintained or even improved their performance on the second day after consolidation in both the OFF and ON stages.

**Table 2:** Domains evaluated and effects found in each article.

Domain	STN-DBS cognitive effects	Articles by
Global Cognitive Functioning/Reasoning	↑ (MMSE)	Lueken et al., 2008; Wouwe et al., 2011
	↑ (DemTect)	Markser et al., 2015
	↑ Addenbrooke	Krishnan et al., 2016
	= (MDRS)	Klempírova et al., 2007; Witt et al., 2008; Daniels et al., 2010
	= (MMSE)	Cilia et al., 2007; Heo et al., 2008; Zanglagia et al., 2009; Israeli-Korn et al., 2013; Asahi et al., 2014
	= (Repeatable Battery For The Assessment of Neuropsychological Status)	Asahi et al., 2014
	= (Wechsler Adult Intelligence Scale)	Asahi et al., 2014
	= (Japanese Adult Reading Test)	Asahi et al., 2014
	= abstract reasoning (Raven Colour Matrices)	Castelli et al., 2007; Castelli et al., 2010; Rizzone et al., 2014
	= (MoCA*)	Tang et al., 2015; Tremblay et al., 2015
	↓ MMSE	York et al., 2009; Kim et al., 2013; Markser et al., 2015; Krishnan et al., 2016
	↓ MDRS	Williams et al., 2010; York et al., 2009; Markser et al., 2015
	↓ Raven	Fasano et al., 2010
Memory	↑ episodic verbal memory (RAVLT* immediate recall)	Tang et al., 2015
	= verbal memory (RKMB)	Heo et al., 2008
	= verbal memory (Benton)	Tang et al., 2015
	= verbal memory (RAVLT*)	Witt et al., 2008; Daniels et al., 2010
	= verbal memory (Bi-syllabic Words Repetition test)	Castelli et al., 2007; Castelli et al., 2010
	= short-term spatial memory (Corsi's Block Tapping Test)	Castelli et al., 2007; Castelli et al., 2010
	= verbal learning (WMS Paired Associate Learning)	Castelli et al., 2007; Castelli et al., 2010
	= memory (Verbal and Digits Span)	Zanglagia et al., 2009
	= memory (MDRS)	Witt et al., 2008
	= memory recognition (verbal and visual Recognition Test)	Yagüez et al., 2014
	↓ immediate, delayed and recognition memory (WMS logical memory)	Klempírova et al., 2007
	↓ delayed memory (RBANS)	Asahi et al., 2014
	↓ episodic verbal memory (RAVLT* immediate and delayed recall)	Fasano et al., 2010; Rizzone et al., 2014
	↓ verbal memory recall (BMPI immediate and delayed memory and learning)	Yagüez et al., 2014
	↓ verbal recognition memory and delayed memory (RKMB)	Heo et al., 2008
Executive Functions	↑ (WCST right categories, right answers, errors nº and non-perseverative errors)	Lueken et al., 2008
	↑ (Stroop effect)	Houvenaghel et al., 2015
	↑ executive functioning (BRIEF-A)	Pham et al., 2015
	↑ visuospatial working memory (working memory tasks)	Ventre-Dominey et al., 2016
	↑ stimulus-action-reward association (Haruno and Kawato task)	Wouwe et al., 2011
	= behavior regulation (BRIEF-A)	Pham et al., 2015
	= semantic verbal fluency (tasks)	Castelli et al., 2007; Castelli et al., 2010; Tremblay et al., 2015
	= phonemic verbal fluency (tasks)	Cilia et al., 2007
	= cognitive flexibility (alternate verbal fluency tasks)	Tremblay et al., 2015
	= cognitive flexibility (Trail Making Test B)	Castelli et al., 2007
	= abstract concept development and cognitive flexibility (WCST*)	Cilia et al., 2007; Castelli et al., 2007; Castelli et al., 2010; Houvenaghel et al., 2015
	= abstract concept development (metaphor comprehension)	Tremblay et al., 2015
	= response initiation and response inhibition (Hayling Sentence Completion Test)	Yagüez et al., 2014
	= working memory (Digit Span)	Daniels et al., 2010; Tang et al., 2015
	= (Stroop effect)	Tang et al., 2015
	= executive functions (Frontal Assessment Battery)	Israeli-Korn et al., 2013
	= processing of outcome errors (Haruno and Kawato task)	Wouwe et al., 2011
	= working memory (n-back DBS STN unilateral task)	Alberts et al., 2008
	↓ logical executive functions (WCST* and Raven)	Zanglagia et al., 2009
	↓ semantic verbal fluency (category task)	Cilia et al., 2007; Witt et al., 2008; York et al., 2009; Daniels et al., 2010; Houvenaghel et al., 2015; Tang et al., 2015; Vonberg et al., 2016
	↓ phonemic verbal fluency (initial recall tasks)	Castelli et al., 2007; Klempírova et al., 2007; Witt et al., 2008; Zanglagia et al., 2009; York et al., 2009; Daniels et al., 2010; Castelli et al., 2010; Fasano et al., 2010; Kim et al., 2013; Yagüez et al., 2014; Houvenaghel et al., 2015; Vonberg et al., 2016
	↓ phonemic and semantic verbal fluency (Delis-Kaplan executive function system)	Williams et al., 2010
	↓ verbal fluency (MDRS Initiative/Perseveration)	Witt et al., 2008
	↓ working memory (Digit Span)	Witt et al., 2008
	↓ cognitive flexibility (WCST* perseverative responses and errors)	Heo et al., 2008; Lueken et al., 2008; Fasano et al., 2010; Rizzone et al., 2014
	↓ (Stroop effect)	Klempírova et al., 2007; Heo et al., 2008; Kim et al., 2013
	↓ interference (Trail Making Test B form)	Klempírova et al., 2007; Kim et al., 2013; Rizzone et al., 2014; Houvenaghel et al., 2015
	↓ planning skills (London Tower)	Klempírova et al., 2007
	↓ working memory (n-back DBS STN bilateral tasks	Alberts et al., 2008
Attention	= (Trail Making Test A and B forms)	Heo et al., 2008; Castelli et al., 2010; Houvenaghel et al., 2015
	= (Stroop)	Witt et al., 2008; Daniels et al., 2010; Tang et al., 2015
	= (MDRS attention)	Witt et al., 2008;
	= (WCST*)	Castelli et al., 2010
	= (Digit Span)	Tang et al., 2015
	↓ (WCST*)	Rizzone et al., 2014
	↓ (RBANS)	Asahi et al., 2014
Perception	= (Visual Object and Space Perception Battery Object Decision task)	Yagüez et al., 2014
	= (Visual Object and Space Perception Incomplete Letters task)	Yagüez et al., 2014
Language	↑ lexical changing and word production	Vonberg et al., 2016
	= (Boston Naming Test*)	Heo et al., 2008; Tang et al., 2015
	= (Graded Naming Test)	Yagüez et al., 2014
	= (lexical decision task and words association task)	Tremblay et al., 2015
	↓ word production	Cilia et al., 2007
	↓ Vocabulary (WASI)	Williams et al., 2010
	↓ fluency tasks (Phonemic and Semantic Verbal Fluency Test)	Rizzone et al., 2014
Visuoconstructive and visuospatial skills	= (MDRS)	Witt et al., 2008
	= visuospatial reasoning (Raven)	Cilia et al., 2007
	= (Benton)	Witt et al., 2008
	= visuospatial organization capacity (Hooper Test)	Tang et al., 2015
	↓ (Corsi's Block Tapping Test forward and backward)	Rizzone et al., 2014
	↓ visuoconstructive skills (RBANS)	Asahi et al., 2014
Motor and sensory coordination	↑ fine motor dexterity and speed (Purdue Pegboard Test)	Wouwe et al., 2011
	= motor and sensory coordination (Purdue Pegboard Test)	Heo et al., 2008
	= (Trail Making Test A)	Klempírova et al., 2007
	= (Stroop Test)	Klempírova et al., 2007

* Indicates different types of versions;

↑indicates "improvement"; = indicates "no changes"; ↓ indicates "decline"; MMSE: Mini-Mental State Exam; MDRS: Mattis Dementia Rating Scale; Raven: Raven Colour Matrices; MoCA: Montreal Cognitive Assessment; RAVLT: Rey Auditory Verbal Learning Test; WMS: Wechsler Memory Scale; RBANS: Repeatable Battery for the Assessment of Neuropsychological Status; BMPI: Birt Memory and Information Processing Battery; RKMB: Rey-Kim Memory Battery; BRIEF-A: Behavior Rating Inventory of Executive Function - Adult Version; STN-DBS: Subthalamic Nucleus Deep Brain Stimulation; WASI: Wechsler Abbreviated Scale of Intelligence.

There were 27 studies involving a total of 832 patients with STN-DBS and 458 patients with DBS and/or healthy subjects in the control group who did not undergo surgery. Age ranged from 51 to 67 years, disease duration ranged from 9.7 to 15.75 years, education (when reported) ranged from 1.9 to 14.5 years, while pre-surgical evaluation occurred 2 weeks before surgery and postoperative up to 132 months after surgery (11 years).

### Global cognitive functioning

Most studies^9^^-^ 21 evaluated global cognitive functioning with 3 different instruments and observed no significant change in subject performance. Only 3 articles^22^^-^ 24 reported impairment in the overall cognitive functioning of their sample.

### Memory

Memory,^10^^,^ 14 as well as specific aspects such as verbal memory,^10^^,^ 11 ^,^ 13 ^,^ 17 ^,^ 18 ^,^ 20 verbal learning,^17^^,^ 18 recognition^27^ and spatial memory,^17^^,^ 18 showed no significant difference before and after surgery, although there was a decline in specific aspects in 6 articles.^9^^,^ 13 ^,^ 16 ^,^ 19 ^,^ 23 ^,^ 27

### Executive function

Several EF aspects were evaluated: visuospatial working memory, stimulus-action-reward association, behavior regulation, semantic and phonemic verbal fluency, cognitive flexibility, abstract concept development, initiation and inhibition responses and working memory. Thirteen articles 11 ^,^ 12 ^,^ 15 ^,^ 17 ^,^ 18 ^,^ 20 ^,^ 21 ^,^ 25 ^-^ 30 reported patient stability or improvement in their results. However, the results presented by the majority of articles^9^^-^ 14 ^,^ 17 ^-^ 19 ^,^ 22 ^-^ 25 ^,^ 27 ^,^ 28 ^,^ 31 ^-^ 35 were the opposite to those observed above, in verbal semantic and phonemic verbal fluency, working memory, planning and cognitive flexibility. 

### Perception and attention

For these two cognitive functions, only two articles^16^^,^ 19 found cognitive decline after STN-DBS in attention, whereas 7 other articles^10^^,^ 11 ^,^ 13 ^,^ 18 ^,^ 20 ^,^ 27 ^,^ 28 found no significant change.

### Language

Five articles^13^^,^ 20 ^,^ 21 ^,^ 27 ^,^ 33 showed better or stable performance in language, production of words,^12^ semantic and phonemic verbal fluency tasks,^19^ and vocabulary subtest of the Wechsler Abbreviated Scale^24^ postoperatively.

### Visuoconstructive and visuospatial skills

Two articles^16^^,^ 19 reported decline in Visuoconstructive and visuospatial skills, while 3 articles^10^^,^ 12 ^,^ 20 showed no difference pre and post-operatively. 

### Motor and sensory coordination

There was no decline in coordination.^9^^,^ 13 ^,^ 26

## DISCUSSION

This systematic review sought to investigate the cognitive functions most affected by STN-DBS according to studies published in the last 10 years. 

Analysis of the results of all 27 articles revealed no consensus among studies on the effect of this surgery on patients. In most articles that evaluated global cognitive functioning, cognition either improved or did not worsen, a good finding since the technique does not target non-motor symptoms. However, STN-DBS can promote an improvement in cognition indirectly in that, once the subject has reduced or eliminated motor symptoms, their quality of life (QoL) improves, allowing them to return to previously discontinued tasks and habits. This behavioral change can yield both cognitive and mood benefits. To confirm this hypothesis would require studies comparing mood (anxiety, depression) before and after stimulation. 

In general, this heterogeneity of results can be due to several factors, as discussed below. The aggravation of cognitive disorders can be strongly predicted by neuropsychological tests in the early stage of the disease, with or without timely medical treatment. On average, 25-50% of PD patients develop MCI or dementia or progress from MCI to dementia within 5 years of diagnosis.^36^ Thus, the selection of instruments is of paramount importance and needs to be accompanied by certain precautions. There is no specific protocol defining the most appropriate instruments for this evaluation, but knowing which functions are influenced by PD makes choosing the tests easier. Establishing a protocol to be used by studies and research centers would render it easier to access, understand and compare results, leading to further investigation of the impaired aspects.^37^ Any change indicated by the tests is subtle, as cognitive impairment detected in specialized tests is not commonly reported by patients, caregivers or health professionals. As stated above, QoL assessments in these patients show improvement, even when cognitive impairment is detected. With regard to memory impairment, for example, there are several associated factors, such as the subject’s age, duration of illness, and even executive functioning. In the case of the articles, the recognition memory^9^^,^ 13 and recall^18^^,^ 19 ^,^ 27 ^,^ 28 were impaired and this is observed in the literature, indicating a possible evolution to dementia in PD.^36^ EFs are an umbrella concept that cover several aspects and, consequently, feature as the most evaluated functions and with the most discrepant results. Commonly, these functions appear to be impaired earlier in the disease and are directly associated with daily activities, impacting patient QoL.^38^ Verbal fluency worsened in many studies.^9^^-^ 12 ^,^ 14 ^-^ 16 ^,^ 22 ^-^ 24 ^,^ 27 ^,^ 28 ^,^ 31 ^,^ 35 In fact, worsening on category fluency tasks is the most frequent cognitive sequela reported after STN-DBS. This is in accordance with recent evidence suggesting that the STN is a potent regulator of basal ganglia and thalamocortical limbic and associative circuits. Frontal lobe-related cognitive changes after DBS may be determined by the modulation of these distinct neural networks.^39^ Impairment of visuospatial skills, in which motor involvement is the main criteria, even in the early stages of the disease, is expected in PD - at odds with the fact that only 5 articles evaluated this function.^10^^,^ 12 ^,^ 16 ^,^ 19 ^,^ 20

One of the inclusion criteria was surgery targeting the STN, and this was one of the limitations found in the studies. STNs are considered to produce more cognitive side effects in patients than when electrodes are implanted in the globus pallidus.^39^ Patient age ranged from 51 to 67 years at the time of surgery and the literature indicates a higher risk of cognitive decline associated with older age. The medication or stimulation parameters in study participants were not controlled, and there may be an influence of reductions in postoperative medication or differences in DBS parameters. On top of this, there are differences regarding follow-ups, making it difficult to understand and establish “specific milestones”, with which improvement or worsening of effects over months/years can be predicted. Thus, while certain articles reported follow-up effects for 36,^22^^,^ 24 84^22^^,^ 26 or up to 132^19^ months, others had data for 12,^24^^,^ 26 6^13^^,^ 15 ^,^ 20 ^,^ 24 and up to 3^17^^,^ 22 ^,^ 31 months. This discrepancy makes a fair comparison and reliable analysis of the data unfeasible. Using the same battery of tests at such widely varying time intervals may give the impression of an improvement simply by the learning effect of a short-term reassessment and a marked worsening as the disease progresses naturally over a long-term reassessment.^37^

There was an absence of reports on the subjective impact of daily cognitive decline associated with motor symptoms^28^ and of preoperative follow-up on cognitive function.^33^ There were no other evaluations of impairment to activities of daily living associated with the disease, which interferes with the subjective perspective of patient abilities. These aspects are directly influenced when motor improvement occurs. Thus, from the recovery of skills, new perspectives emerge, which can have a positive repercussion on non-motor symptoms, such as cognition. The angle of the surgical trajectory and proximity of the STN-DBS electrodes greatly influences the outcomes seen after surgery, where these aspects may be related to changes in the cognitive and emotional functioning of patients.^12^^,^ 33 Thus, the results are expected to vary from one another - as has been seen. This disparity is mainly due to variations in the characteristics of patients selected for surgery across different centers (age,^21^^,^ 26 preoperative state^10^^,^ 24 and comorbidity with other conditions such as psychiatric disorders^11^^,^ 12), making conclusions difficult to compare and analyze.

Thus, it was not possible to establish a neuropsychological profile of PD patients with STN-DBS. This is cause for concern since patients with MCI in PD are more likely to progress to dementia as the disease progresses, and it is necessary to understand which cognitive functions become impaired in this disease after DBS implantation to avoid miscalculating normal with worsening evolution. Much of this can also be attributed to the lack of a specific PD assessment protocol.^37^

The results of this review highlight the need to establish a neuropsychological profile of PD patients to understand and investigate the effects of implantation of STN-DBS on cognitive non-motor symptoms. Future studies intend to develop a neuropsychological battery and evaluate patients with PD and STN-DBS to discriminate the aspects affected in these subjects and understand which factors contribute to outcomes.
